# Impact of Molecular and Cytogenetic Responses on Long-Term Outcomes in Children and Adolescents With Chronic Myeloid Leukemia: A Retrospective Study From India

**DOI:** 10.7759/cureus.89359

**Published:** 2025-08-04

**Authors:** Dipesh Dave, Maharshi Trivedi, Chinmay Doctor, Biren Parikh, Harsha P Panchal, Rajan Yadav

**Affiliations:** 1 Pediatric Oncology, The Gujarat Cancer and Research Institute, Ahmedabad, IND; 2 Oncopathology, The Gujarat Cancer and Research Institute, Ahmedabad, IND; 3 Medical Oncology, The Gujarat Cancer and Research Institute, Ahmedabad, IND

**Keywords:** cytogenetic response, indian data on pediatric cml, long-term outcomes, molecular response, pediatric cml, pediatric oncology

## Abstract

Introduction

Chronic myelogenous leukemia (CML) in pediatric and adolescent populations is relatively rare. The present study provides an integrated approach to evaluate the impact of molecular and cytogenetic response on long-term outcomes in these populations by incorporating demographic factors and hematological parameters, and to explore their clinical relevance in resource-limited settings.

Material and methods

A retrospective analysis was conducted on patients <18 years with newly diagnosed CML from January 2014 to December 2023 at the Gujarat Cancer and Research Institute in India. Data were retrieved from the hospital database, including demographics, clinical details, hematological parameters, CML phase at diagnosis, and cytogenetic and molecular studies. Of 73 diagnosed patients, eighteen were not subjected to molecular, cytogenetic, and complete hematological response (CHR) analysis due to factors such as irregular follow-up, testing constraints, and financial or logistic barriers.

Results

Of the 73 patients, seventeen (23%) were below 10 years of age, and 56 (77%) were above 10 years at the time of diagnosis. The male-to-female ratio was 1.8:1. Seventy (96%) of the 73 patients were initially diagnosed with CML in the chronic phase, and three patients (4%) were diagnosed with CML in blast crisis. Response evaluation was conducted on 55 patients. Of these, CHR was observed in 41 (74%) patients at 3 months. At 12 months, forty (73%) achieved complete cytogenetic response (CCyR), and 25 (45%) achieved major molecular response (MMR). Among the 55 patients, those who achieved CCyR and MMR at 12 months had a 100% five-year survival, compared to 66.7% and 83%, respectively, in those who did not achieve a response. Patients who achieved early molecular response (EMR) had a 97.4% five-year survival. The Kaplan-Meier estimated overall survival was 91.6% at 3 years and 87.6% at 5 years. Fifty of the 55 patients (91%) were alive at long-term follow-up, with a median duration of follow-up of 62 months (range: 0-128 months). Patients aged 0-10 years and >10 years had five-year survival rates of 100% and 88%, respectively.

Conclusion

The achievement of molecular and cytogenetic responses has a significant impact on long-term outcomes in pediatric and adolescent patients diagnosed with CML, highlighting their role as crucial predictors in clinical practice. Patients who achieved both MMR and CCyR demonstrated 100% five-year overall survival (OS), while those with EMR showed a 97.4% five-year survival. These findings support the utility of early response monitoring to inform prognosis and guide therapy, particularly in low- and middle-income countries (LMICs). However, treatment abandonment and poor compliance remain major obstacles in LMICs. Further research with multicentric prospective trials in LMICs is essential.

## Introduction

Background

Chronic myeloid leukemia (CML) is a myeloproliferative hematological malignancy derived from an abnormal pluripotent bone marrow stem cell. CML is rare in pediatric and adolescent populations, constituting approximately 2%-3% of leukemia cases, with a median age of 11 to 12 years (range 1-18 years) at diagnosis [1-4]. The Philadelphia (Ph) chromosome, created by a translocation between chromosomes 9 and 22, generates the Breakpoint Cluster Region-Abelson leukemia virus (BCR-ABL), which leads to the abnormal protein BCR-ABL1 tyrosine kinase, central to the development of CML [3]. This distinctive protein provides a target for tyrosine kinase inhibitors, especially imatinib mesylate (IM), which has drastically improved outcomes in CML [3].

Challenges in low- and middle-income countries

Despite excellent outcomes in high-income countries, treating pediatric and adolescent CML in low- and middle-income countries (LMICs) remains challenging, and the prognosis is not as good due to delayed diagnosis, late presentation, financial constraints, limited access to care, and treatment abandonment [1].

Study objectives

Prognostic scoring systems such as Sokal, EURO, EUTOS, and Hasford are widely applied in adult CML management [5,6]. In adult CML patients, early molecular and cytogenetic responses are well-established predictors of long-term survival [7]. However, in pediatric CML, data on such evaluations remain poorly defined, and no risk stratification tool has been specifically developed for this age group. As a result, management in these populations often relies on adult guidelines despite unique disease biology. Therefore, the objective of this study is to characterize pediatric and adolescent patients diagnosed with CML, analyze the association between molecular and cytogenetic responses and outcomes, and identify predictors of treatment success to guide future implementation strategies in LMICs.

## Materials and methods

Patient selection and study design

A retrospective study was conducted at Gujarat Cancer and Research Institute, Ahmedabad, India, from January 2014 to December 2023. Prior to data collection, approval was obtained from the institutional review committee (IRC/2025/P-03). Children and adolescents aged <18 years newly diagnosed with CML according to WHO criteria were included in the study [8]. Demographic and clinical details were collected from the hospital database. Diagnostic investigations included bone marrow aspiration for morphological assessment, flow cytometry, fluorescent in-situ hybridization (FISH) for t(9;22) translocation, and imaging modalities such as abdominal ultrasound and chest X-ray, which were routinely performed in all patients. Hematological parameters, BCR-ABL molecular response, and cytogenetic responses were collected at diagnosis and at 3, 6, and 12 months. Patients who abandoned treatment during the year, had financial constraints that precluded molecular testing, or exhibited poor drug compliance were not evaluated for hematological, molecular, and cytogenetic responses. Advanced molecular studies, such as BCR-ABL1 tyrosine kinase domain (TKD) mutation testing and imatinib resistance mutation analysis (IRMA), were not available at our institution due to the lack of advanced diagnostic facilities. However, IRMA testing was performed in selected patients, where feasible, through accredited reference laboratories, in line with standardized protocols to ensure reliability.

Treatment protocols

For CML chronic phase (CML-CP), imatinib mesylate (IM) was administered at 260-340 mg/m²/day, and dasatinib at 60 mg/m²/day depending on availability [9]. Other second-generation tyrosine kinase inhibitors (2GTKIs) were unavailable in our institution. For CML blast crisis (CML-BC) phase, IM and dasatinib were given at 340 mg/m²/day and 80 mg/m²/day, respectively, along with the appropriate BC leukemia regimen based on the blast cell type, followed by hematopoietic stem cell transplantation (HSCT) if eligible [10,11].

Response assessment

Complete hematological response (CHR) was defined as a WBC count <10 × 10⁹/L, platelet count <450 × 10⁹/L, normal differential with no early forms, and no splenomegaly [4]. Complete cytogenetic response (CCyR) was defined as no Ph-positive metaphases on karyotyping. A BCR-ABL ratio ≤1% on the international scale (IS) by quantitative reverse transcription polymerase chain reaction (RT-PCR) was considered equivalent to CCyR [12]. Early molecular response (EMR) was defined as a BCR-ABL ratio ≤10% at 3 months. A major molecular response (MMR) was defined as BCR-ABL1 IS transcripts ≤0.1%. Molecular responses were analyzed based on European LeukemiaNet (ELN) recommendations [12].

Suboptimal response was defined as failure to achieve CHR by three months or CCyR and MMR (BCR-ABL1 >0.1% IS) by 12 months. In patients demonstrating a suboptimal response, adherence and tolerability to IM were first assessed. Where clinically appropriate and well-tolerated, dose escalation of IM was attempted. Patients were closely monitored over the subsequent three-month period to evaluate for delayed treatment response. In cases where no improvement was observed after this interval, IRMA was performed when available, and therapy was switched to dasatinib. Treatment failure was defined as persistent BCR-ABL1 transcript levels >10% at 6 months, >1% at 12 months, or any loss of a previously achieved hematologic, cytogenetic, or molecular response [12]. For patients meeting criteria for treatment failure, assessment included evaluation of medication adherence, IRMA testing when feasible, and prompt transition to a 2GTKI, irrespective of mutation status, if clinically indicated. An event was defined as a loss of hematological response, progression to an advanced phase, or death.

Statistical analysis

All data were encoded in a Microsoft Excel sheet. Descriptive statistics were performed for demographic data. Statistical analysis was conducted using SPSS software version 22.0 (IBM Corp., Armonk, USA). Survival rates were computed using the Kaplan-Meier method. The log-rank test was used to statistically compare survival distributions among different groups. All statistical tests were considered significant at a p-value ≤0.05. A multivariable Cox regression analysis was conducted to evaluate independent prognostic factors for survival. Variables included age, disease phase at diagnosis, hemoglobin, total leukocyte count, platelet count, and treatment modality. Variables were selected based on clinical relevance and data completeness.

## Results

Patient demographics and clinical characteristics

A total of 73 pediatric and adolescent patients were diagnosed with CML during the study period. The median age at diagnosis was 13 years (range: 1-18 years), with a male predominance. The male-to-female ratio was 1.8:1. The baseline demographics along with clinical and hematological details are presented in Table 1. Seventy (96%) of the 73 patients were initially diagnosed with CML-CP, and 3 (4%) were diagnosed with CML-BC phase. None of the patients were diagnosed with CML-AP in this study.

**Table 1:** Baseline clinical and hematological parameters of 73 CML patients. CML: Chronic myelogenous leukemia; Hb: Hemoglobin; CP: Chronic phase; BC: Blast Crisis.

Parameters	Results
Age	
0-10 years	17(23%)
> 10 years	56(77%)
Gender	
Male	47(64%)
Female	26(36%)
Phase of CML	
CP	70 (96%)
BC	3 (4%)
Hb	
<8 gm/dL	30 (41%)
8-10gm/dL	31 (43%)
>10gm/dL	12 (16%)
WBC	
<200 x 10^9^/L	26( 36%)
200-400 x 10^9^/L	24 (33%)
>400 x 10^9^/L	23 (31%)
Platelets	
<450 x 10^9^/L	40 (55%)
450-1000 x 10^9^/L	26 (36%)
>1000 x 10^9^/L	7 (9%)
Symptoms	
Abdominal discomfort	42 (57%)
Low grade fever	37 (51%)
Weakness and Fatigue	33 (45%)
Loss of appetite	33 (45%)
Loss of weight	17 (23%)
Splenomegaly	66 (90%)
Hepatomegaly	37 (51%)
Pallor	28 (38%)

At the time of diagnosis, the mean Hb was 8.51 g/dL (range: 3.8-13.5), the mean WBC count was 318.58 × 10⁹/L (range: 4.10-862.78 × 10⁹/L), and the mean platelet count was 497.26 × 10⁹/L (range: 9-1640 × 10⁹/L). Abdominal discomfort, low-grade fever, weakness and fatigue, and decreased appetite were common symptoms. Splenomegaly and hepatomegaly were observed in 90% and 51% of patients, respectively.

Treatment characteristics

Of the 73 diagnosed patients, long-term hematological and molecular assessments were available for 55 patients. In the remaining 18, data were limited due to factors such as irregular follow-up, testing constraints, and financial or logistical barriers. Among the 55 patients, 46 (84%) received imatinib mesylate (IM) as primary treatment. Of these, five patients later switched to dasatinib due to suboptimal response and/or treatment failure with IM. Two patients progressed to the BC phase (median time: 10 months; range: 6-14 months) and received a BC leukemia regimen along with IM but ultimately succumbed to the disease. Six (11%) patients received dasatinib as the primary treatment due to physician preference. Of the three patients initially diagnosed in the CML-BC phase, one received only the BC leukemia regimen, and another received IM with the BC leukemia regimen; both succumbed to the disease. The third patient underwent HSCT after responding to dasatinib in combination with the BC leukemia regimen and achieving a second chronic phase. The patient was subsequently maintained on dasatinib monotherapy due to high risk of relapse.

Response to treatment and survival analysis

**Figure 1:**
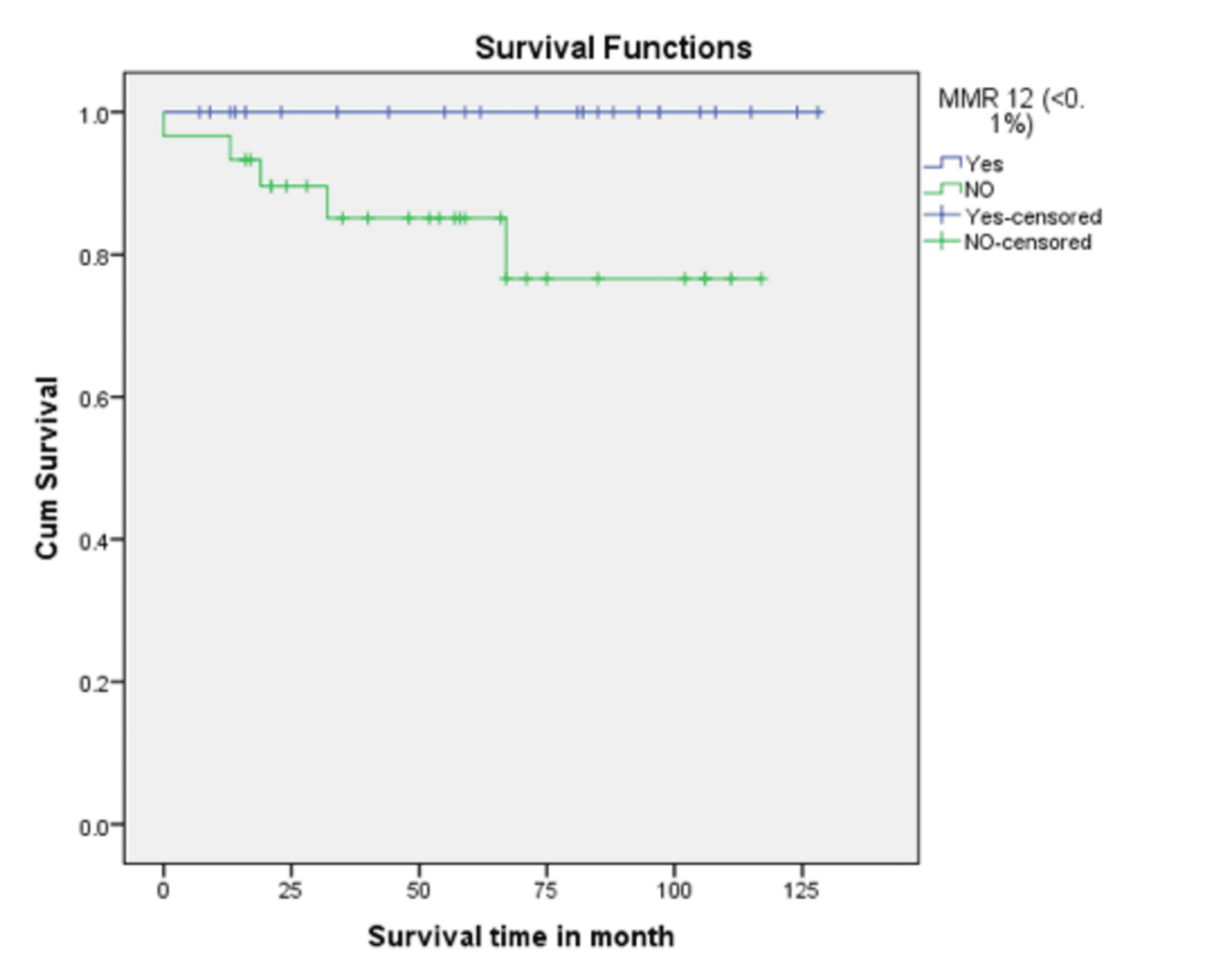
Kaplan-Meier survival curve based on MMR status at 12 months. Patients who achieved MMR at 12 months had a 100% five-year survival rate, compared with 83% in those who did not achieve MMR. MMR 12: Major Molecular Response at 12 months.

**Figure 2:**
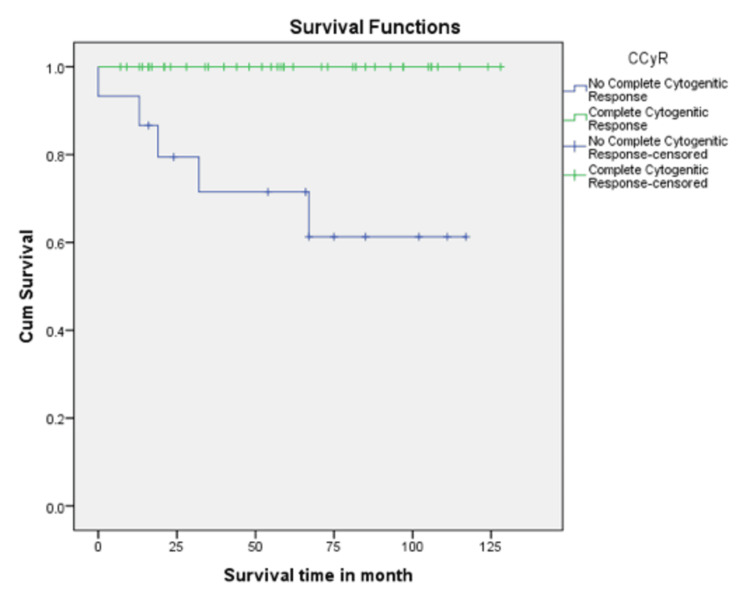
Kaplan-Meier survival curve based on CCyR status at 12 months. Patients who achieved CCyR at 12 months had a 100% five-year survival rate, compared with 66.7% in those who did not achieve CCyR. CCyR: Complete Cytogenetic Response.

**Figure 3:**
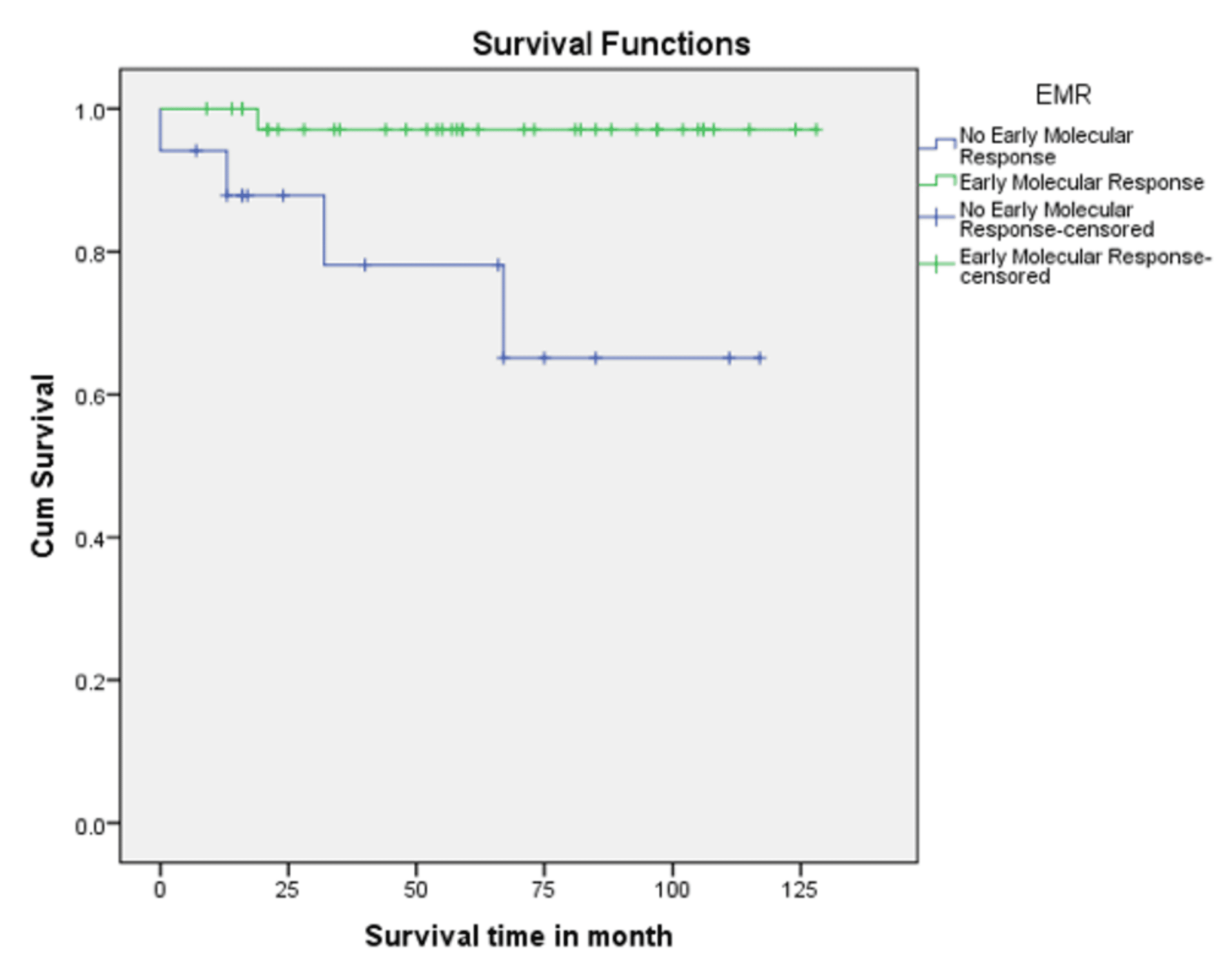
Kaplan-Meier survival curve based on EMR status at three months. Patients who achieved EMR at three months had a 97.4% five-year survival rate, compared with 76.5% in those who did not achieve EMR. EMR: Early Molecular Response.

In this study, the CHR rate at 3 months was 74%. CCyR and MMR at 12 months from diagnosis were achieved in 40 (73%) and 25 (45%) patients, respectively. Patients who achieved MMR at 12 months had 100% five-year survival (p = 0.033), compared with those who did not achieve MMR, who had 83% survival (Figure 1). Patients who achieved CCyR at 12 months had 100% five-year survival (p < 0.001), compared with those who did not, who had 66.7% survival (Figure 2). EMR was achieved in 38 (69%) patients at 3 months. Patients who achieved EMR had a significantly higher five-year survival rate (97.4%) compared to those who did not (76.5%), with a p-value of 0.007 (Figure 3). In the multivariable Cox regression analysis, only the disease phase at presentation (specifically chronic phase) was significantly associated with survival (p = 0.03). Other variables, including age, hemoglobin level, total leukocyte count, and treatment modality, did not demonstrate a statistically significant association with survival.

**Figure 4:**
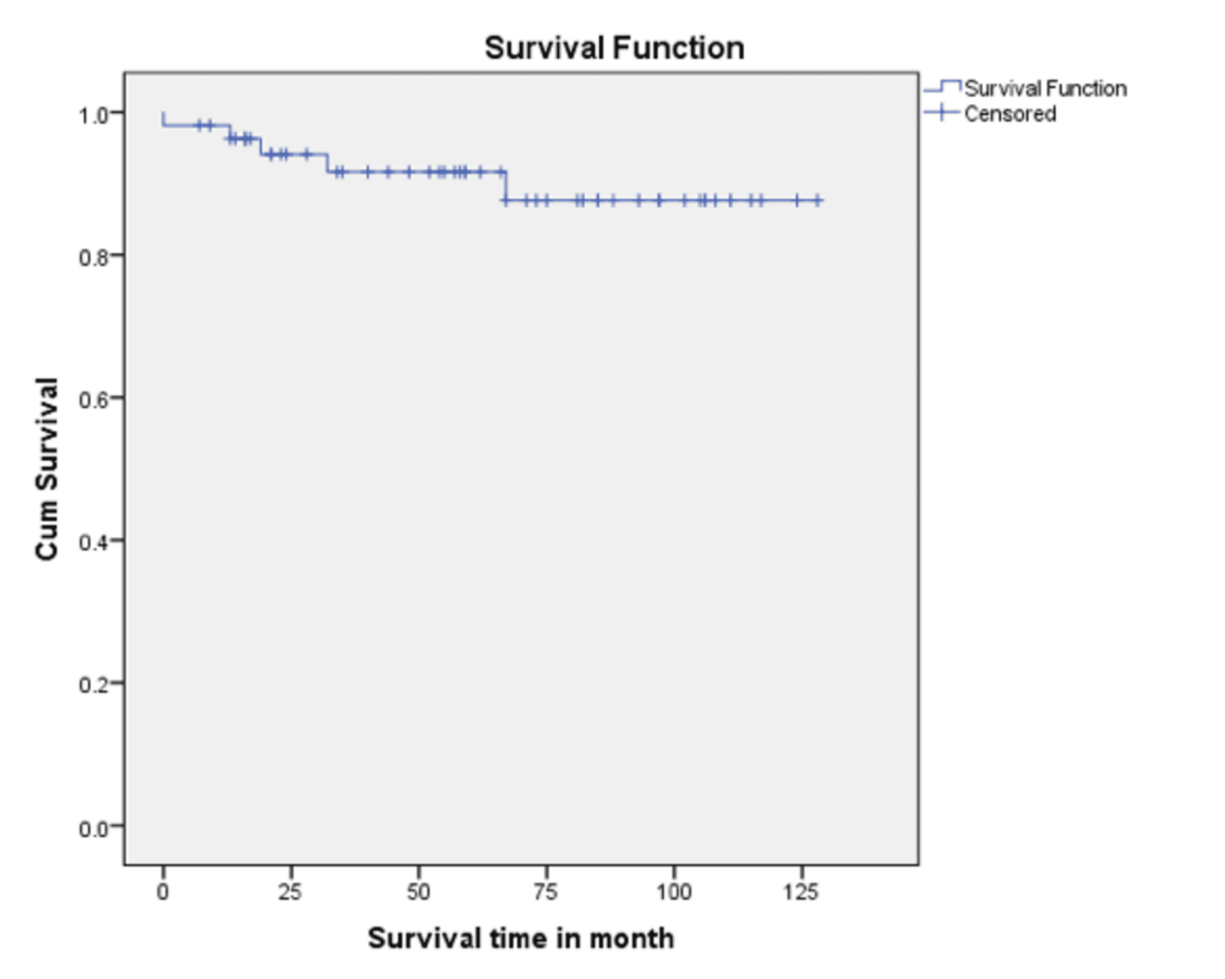
Overall survival curve for the study cohort. The three-year and five-year overall survival (OS) rates were 91.6% and 87.6%, respectively.

At the last follow-up, 50 of the 55 patients (91%) were alive, with a median follow-up duration of 62 months (range: 0-128 months) (Figure 4). The Kaplan-Meier estimated overall survival was 91.6% at 3 years and 87.6% at 5 years. Five patients (9%) died: two due to advanced disease at presentation (i.e., diagnosed in BC phase), and two due to progression from CP to BC phase with loss of hematological response. The median time to event was 31 months (range: 0-74 months). One patient died from causes unrelated to the disease; however, the specific cause was not documented. The five-year survival rate was 100% in patients aged 0-10 years and 88% in those older than 10 years. 

## Discussion

Rarity of pediatric CML

CML is a very rare disease in children, with less than 10% of all CML cases diagnosed in children and adolescents. CML accounts for 3%-5% of all childhood leukemias and has an annual incidence of approximately one in 1,000,000 [13]. A multicentric Phase I study conducted by a children’s oncology group that included 31 children underscored the rarity of CML in this age group. The majority of children and adolescents are diagnosed with CML-CP, with far fewer cases of CML-AP and CML-BC [14,15]. In the present study, 96% of patients presented with CML-CP and 4% with CML-BC. The documented median age at diagnosis was 13 years, with approximately 4% presenting in the advanced CML phase, findings consistent with the present study [14,15].

Hematological differences

Children and adolescents with CML have unique characteristics and different hematological profiles compared to adults [16]. The mean leukocyte count has been reported to be four times higher in pediatric patients than in adults in some studies [3,16,17]. Other studies have also noted higher platelet counts in pediatric cases [5,17]. These findings are consistent with the results of our study.

Prognostic value of molecular and cytogenetic response

Several prognostic scoring systems are available for adult CML; however, no validated prognostic tool currently exists for the pediatric population, making it challenging to predict long-term outcomes in this cohort. In their study, Kesana S et al. highlighted that attaining cytogenetic and molecular responses is a key milestone in predicting long-term outcomes [15]. They reported lower CCyR (35%) and MMR (19%) rates at 12 months, which were associated with a reduced 8-year OS of 80.4%, underscoring the negative impact of delayed or suboptimal response [15]. In the study by Ganguly S et al., although 124 pediatric CML patients were enrolled, only 81 were analyzed for cytogenetic and molecular responses [18]. They reported a CCyR rate of 54% and an MMR rate of 50.9% at 12 months, with a corresponding 5-year OS of 92% ± 3%, supporting the strong link between EMR and long-term survival [18]. In the current study, although only 55 of 73 patients were evaluable for cytogenetic and molecular responses, we observed a higher CCyR rate of 73% and an MMR rate of 45%, which translated into a favorable three-year OS of 91.6% and a 5-year OS of 87.6%. Additionally, multivariable Cox regression was applied to identify independent predictors of survival. Among the clinical and hematological parameters assessed, only the chronic phase at diagnosis showed a statistically significant association with survival (p = 0.03). Taken together, these findings reinforce the prognostic relevance of disease phase at diagnosis and suggest that achieving CCyR and MMR within the first year of therapy may provide a clinical benefit that warrants further investigation in pediatric CML.

EMR and survival outcomes

Studies have reported that EMR at 3 months is a significant predictor of long-term survival [6]. The current study found results consistent with a French Phase 4 study on Glivec involving 40 children with CML, which documented the prognostic significance of EMR on long-term outcomes [19]. In our cohort, a nearly 97% five-year survival rate was observed in patients who achieved EMR at 3 months from the start of treatment.

Potential of molecular and cytogenetic responses as early markers

A five-year survival rate of 100% was observed in patients who achieved both MMR and CCyR. The findings of this study demonstrated a significant association between these response criteria and overall survival in pediatric and adolescent CML, suggesting their potential utility as prognostic markers. Incorporating molecular and cytogenetic response monitoring into routine early follow-up could help identify patients at higher risk for suboptimal outcomes and support timely therapeutic interventions, including dose escalation or switching to second-generation TKIs (2GTKIs), to enhance molecular response and long-term outcomes in this population.

Challenges to molecular response in real-world LMICs

Despite achieving favorable long-term clinical outcomes in this population, hematological and molecular response rates were below expectations, even among patients who regularly attended follow-ups and reported good adherence to treatment. This aligns with findings from a multicentric study in Taiwan, which reported slower molecular responses to front-line IM in pediatric patients, despite similar 10-year OS in real-world practice [20]. Such delays in response may stem from challenges prevalent in LMICs, including poor disease awareness, long travel distances to healthcare centers, illiteracy, and limited understanding of the importance of long-term medication adherence. Slower responses may also result from intermittent noncompliance when patients and caregivers mistakenly perceive the disease to be resolved following symptomatic improvement. These factors may contribute to disease progression despite apparent long-term disease control [21,22].

Strategies for LMICs

To improve outcomes in LMICs, future strategies should focus on enhancing access to early diagnosis, supporting treatment adherence, and ensuring consistent drug availability. The development of tailored, context-specific treatment guidelines and investment in diagnostic infrastructure are critical. Regional collaboration through registries and shared data platforms can help track outcomes, inform policy, and support equitable cancer-care reforms.

Strengths and limitations

The major strength of this study is its demonstration of the real-world relevance of molecular and cytogenetic responses in relation to overall survival in LMIC settings, supported by a long duration of follow-up in pediatric and adolescent CML patients. Although preliminary, these findings provide a foundation for further validation through larger, multicenter prospective studies. However, this study also has limitations, including its single-center, retrospective design, small sample size, limited access to advanced diagnostic testing, therapeutic inequities, and issues with treatment compliance.

## Conclusions

The low incidence of pediatric CML limits clinical experience and hinders the development of standardized treatment protocols. Despite the challenges faced in LMICs, favorable outcomes are achievable with appropriate therapy and consistent monitoring. Early achievement of EMR, CCyR, and MMR is associated with improved long-term outcomes and may serve as practical indicators to guide therapeutic decisions. Multicenter prospective studies in LMIC settings are essential to generate robust evidence and support the development of pediatric-specific treatment strategies.
